# Event Stream Denoising Method Based on Spatio-Temporal Density and Time Sequence Analysis

**DOI:** 10.3390/s24206527

**Published:** 2024-10-10

**Authors:** Haiyan Jiang, Xiaoshuang Wang, Wei Tang, Qinghui Song, Qingjun Song, Wenchao Hao

**Affiliations:** College of Intelligent Equipment, Shandong University of Science and Technology, Tai’an 271000, China

**Keywords:** event camera, event stream visualization, denoising

## Abstract

An event camera is a neuromimetic sensor inspired by the human retinal imaging principle, which has the advantages of high dynamic range, high temporal resolution, and low power consumption. Due to the interference of hardware and software and other factors, the event stream output from the event camera usually contains a large amount of noise, and traditional denoising algorithms cannot be applied to the event stream. To better deal with different kinds of noise and enhance the robustness of the denoising algorithm, based on the spatio-temporal distribution characteristics of effective events and noise, an event stream noise reduction and visualization algorithm is proposed. The event stream enters fine filtering after filtering the BA noise based on spatio-temporal density. The fine filtering performs time sequence analysis on the event pixels and the neighboring pixels to filter out hot noise. The proposed visualization algorithm adaptively overlaps the events of the previous frame according to the event density difference to obtain clear and coherent event frames. We conducted denoising and visualization experiments on real scenes and public datasets, respectively, and the experiments show that our algorithm is effective in filtering noise and obtaining clear and coherent event frames under different event stream densities and noise backgrounds.

## 1. Introduction

An event camera is an emerging bio-visual sensor that records data based on light intensity changes at each pixel point [1,2]. When the light intensity change exceeds a preset threshold, the event camera asynchronously outputs the pixel address and timestamp to form the event stream data. In scenarios such as strenuous motion, changing lighting conditions, or platform power constraints, the motion blur and exposure problems faced by traditional frame cameras can significantly reduce the detection of valid feature points, thus decreasing the reliability of the visual information, while event cameras can effectively compensate for these shortcomings and provide more stable and reliable visual data. The unique nature of event cameras allows them to show significant advantages in several fields, such as image reconstruction [3,4,5,6,7], 3D reconstruction [8,9,10,11,12], optical flow [13,14,15,16], and target tracking [17,18,19,20]. In addition, event cameras show potential applications in visual navigation and localization [21,22,23].

**Figure 1:**
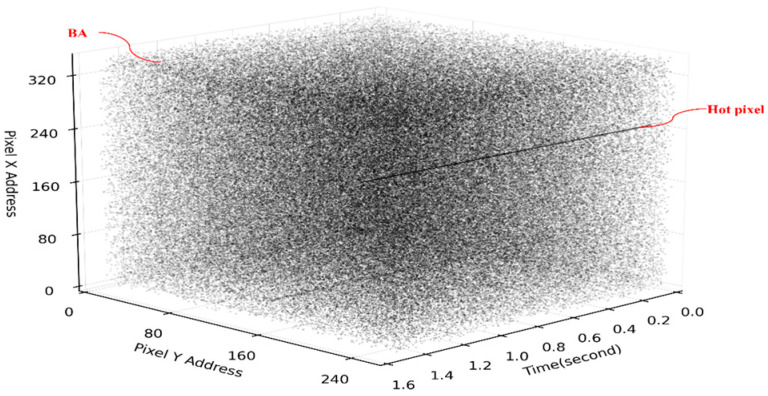
Random distribution for BA noise and a straight line over time for hot noise.

There are many challenges faced by event cameras in practical applications. For example, changes in the camera motion state and in the scene mean the event stream is not uniformly distributed over different periods, making it difficult to obtain clear and informative coherent event frames with traditional event stream visualization methods. Due to the high sensitivity of event cameras to changes in ambient brightness and junction leakage currents, there is an output even if there is no change in light intensity, called background activity (BA) [24], which affects the image quality, wastes communication bandwidth, and consumes unnecessary computational power. In high-speed operating environments and long exposures, event cameras experience hot pixels similar to conventional image sensors [25], where some pixels produce abnormal signals or offsets, which can cause these pixels to fail to reset correctly and continue to output events, called hot noise. Asynchronous events in an event camera capturing a rapidly changing scene are subject to a large amount of noise, which degrades the quality of the event data, blurs the event information in the event stream, and may even mask the true event signals. Traditional graph and video denoising methods are difficult to directly apply to event stream denoising, so there is an urgent need for efficient and feasible event stream denoising methods. Figure 1 shows the spatio-temporal visualization of the hot pixel noise and the background activity noise in a stream of events, with the X and Y axes representing the horizontal and vertical coordinates of the events, and the Taxis coordinates corresponding to the timestamps of the events. From the figure, it can be seen that the hot noise is parallel to the time axis and occurs continuously at a single pixel; the BA noise distribution is more random and sparser from the visualization.

Although event cameras work differently from traditional frame cameras, event stream data are still affected by various noises, such as sensor noise, ambient light changes, motion blur, etc. Therefore, event stream denoising is still a challenging problem.

In this paper, we propose an event stream denoising method based on spatio-temporal correlation differences. The design philosophy of the proposed method is as follows. The regular motion of objects generates a large number of events in the event camera, which are regarded as real events. However, due to the impact of the event camera hardware and other factors, a significant amount of noise is also present in the event stream. This noise can be categorized into BA noise and hot noise based on their spatio-temporal distribution characteristics. We propose a cascade filtering method for denoising these two types of noise based on their spatio-temporal distribution properties. Firstly, the event stream undergoes coarse filtering based on spatio-temporal density to remove BA noise, which exhibits sparsity in the spatiotemporal domain, and then proceeds to fine filtering. Secondly, leveraging the high temporal resolution of the event camera, we treat the event stream generated by each pixel within each time segment as a time series. To address the characteristic of hot noise, which exhibits a fixed high-frequency pattern in the spatiotemporal domain, we propose a time sequence analysis method to filter out hot noise. Our method primarily aims to enhance the robustness of denoising across various noise environments. Therefore, in the fine filtering stage, we also consider the impact of residual BA noise. Furthermore, we propose an adaptive overlap-based event stream visualization method, which adjusts the number of events from the previous frame that are overlapped onto the current frame based on the overlapping strategy. The main contributions of this work can be summarized as follows.

- An event stream denoising method based on spatio-temporal density and temporal sequence analysis is proposed. The denoising process is divided into coarse filtering and fine filtering, and after establishing the spatio-temporal neighborhood of events, the noise is removed sequentially according to the spatio-temporal distribution characteristics of different noises.
- An adaptive overlap-based event stream visualization method is proposed for the event stream density change over time, which dynamically adjusts the overlap time according to the change of the event density of neighboring frames, and obtains clear and information-coherent event frames for further event camera-based research.

## 2. Related Work

In this section, we present and discuss the classical event stream noise reduction methods and event stream visualization methods.

### 2.1. Existing Event Stream Noise Reduction Algorithms

In recent years, a large number of scholars have conducted in-depth research on event camera denoising, and the event stream denoising method based on spatio-temporal correlation is one of the main research directions, which removes noise by targeting the regular difference between noise and real events in time and space. Wu et al. [26] introduced a denoising method based on a probabilistic undirected graph model, proposed a priori knowledge based on the working principle of DVS and the object motion law, constructed a probabilistic undirected graph model, transformed the event stream denoising problem into a model energy minimization optimization problem, and optimized the model by using the improved ICM to obtain the denoised event stream. The model is built based on the distribution characteristics of BA noise, which does not take into account the influence of hot noise. Feng et al. [27] proposed a denoising method based on event density. Firstly, spatio-temporal neighborhood and event density matrices are built using coarse filtering, and the BA noise is removed by calculating the L1 paradigm number of the matrix. The valid events are stored in the coarse filtering results, then the event density matrices are established and the scintillation noise judgment is used to filter out the hot noise using the flicker noise determination value R.

Khodamorad et al. [28] proposed a novel spatio-temporal filter with memory complexity that stores fewer timestamps in a specific way based on the sparse random nature of BA noise. This allocates two memory cells per row and column to store the latest event in the whole row or column, and if two events are captured in the same but different rows in a short window of time, the most recent event overwrites the old event in memory. The complexity of the filter created based on the new storage approach is reduced from $O(N^{2})$ to O(N), which improves memory utilization. However, it is difficult to remove dense noise using a single spatio-temporal filter.

To better utilize the spatio-temporal features, some scholars introduced neural networks to improve the event stream denoising performance. Event-denoising neural networks can be categorized into three types according to their input: 3D vector input, image input, and asynchronous event input. Ryumin et al. [29] proposed a research methodology for audio-visual speech recognition (AVSR) in driver assistance systems. Their spatio-temporal fusion strategy leverages contextual information from both the spatial and temporal domains, preserving the contextual integrity of both modalities while ensuring their synchronization. Arindam et al. [30] proposed a new spike neural network-based method NN2 for filtering noise events from data captured by asynchronous time-based image sensors on the IBM TrueNorth neural synapse system, a neural morphology processor. Fang et al. [31] proposed an event-based deep learning approach for the event denoising method AEDNet, which directly utilizes the raw DVS data from the event stream without destroying the inherent spatio-temporal correlation. The temporal and spatial information is processed separately using temporal window and spatial feature embedding modules, which decompose the events into temporal correlation and spatial affinity and process these two parts separately to remove the noise. In addition, the event frames obtained from the transformation of asynchronous event streams are denoised using a conventional image-denoising algorithm. Xie et al. proposed an image-based denoising method by visualizing the event stream to obtain event frames, which are subsequently denoised using traditional K-SVD [32], but this method loses the temporal attributes of the data, which seriously affects the high-speed characteristics of the event camera.

Overall, most existing event stream denoising methods only focus on filtering out random noise, ignoring the impact of hot noise or not considering the robustness of denoising algorithms in dense noise environments. Although simple algorithm designs may improve denoising efficiency, the accuracy of denoising may decrease, seriously affecting the further use of event streams.

### 2.2. Existing Approaches to Event Stream Visualization

**Figure 2:**
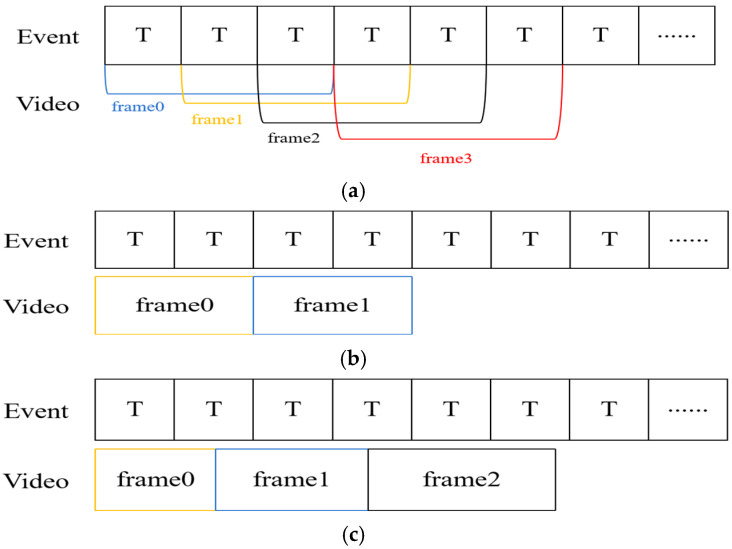
Event stream visualization methods for (**a**) overlapping accumulation, (**b**) fixed time intervals, and (**c**) fixed number of events.

Asynchronous event stream information form is difficult to process, and a single event contains too little information, so the processing needs to aggregate events for processing. In addition, the event stream information is not intuitive for human eyes to observe, so it needs to be visualized to advance to the next step. Related work based on event camera visualization can be briefly divided into two categories: 1. For the event stream itself, through a fixed time window and other ways to obtain the event frame, which is convenient for further observation and application processing; and 2. image reconstruction of the event stream information, to obtain the estimated intensity of the image to achieve the effect of visualization. Xie et al. [33] proposed an overlap accumulation-based method to convert events with periods from 0 ms to 30 ms to frame 0 and events with periods from 10 ms to 40 ms to frame 1, as shown in Figure 2a. Although it makes the event information very rich, it also brings the problem of information redundancy or incoherent information in event frames, which is not conducive to real-time detection and monitoring. Chen et al. [34] proposed an event stream visualization scheme to achieve high and low frame rate adjustment and verified its effectiveness on the FPGA platform. However, the method reduces the amount of information contained in the event frames at high frame rates, and the information obtained during smooth motion is too little for further processing. In addition, the traditional fixed time interval approach divides the event stream into multiple sub-segments according to a fixed time window starting from the timestamp of the first event, and all events within each sub-segment are accumulated and projected to the event frame at the end of that sub-segment as shown in Figure 2b. The fixed number of events approach divides the event stream into multiple sub-segments by counting every N event starting from the first event, and all events within each sub-segment are accumulated and projected to the event frame at the end of the sub-segment, as shown in Figure 2c.

## 3. Event Stream Denoising and Visualization Method

We proposed an event stream noise reduction and visualization algorithm. Based on the noise distribution characteristics, the event stream noise is categorized into BA noise and hot noise, and the event stream is sequentially coarse filtered and fine filtered. In this paper, we pay more attention to the optimization and improvement of the robustness of the denoising algorithm, so the parameter selection and the influence of the residual BA noise are taken into account in different filtering stages. The spatio-temporal neighborhood regularization and the free choice of threshold make our algorithm applicable to various noise environments. The visualization algorithm adaptively overlaps the events of the previous frame according to the change in event density.

### 3.1. Representation of Event Data

Event cameras asynchronously generate data that exhibits a sparse distribution in space. Typically, the event camera outputs events containing the pixel address, time, and type of event. The event stream is defined as $\left\{e_{i}\right\}$, where each event $e_{i}=(t_{i},x_{i},y_{i},p_{i})$ contains the coordinates of the event’s position on the pixel plane $\left(x_{i},y_{i}\right)$, the timestamp of the event’s occurrence $t_{i}$, and the polarity of the event $p_{i}$.

The polarity of an event is determined by the change in light intensity. If at time $t_{i}$, the luminance of a pixel point increases compared to the previous moment and this increase exceeds a predetermined threshold, the event is recorded as a positive polarity event $p_{i}=+1$; conversely, if the luminance decreases and exceeds the threshold, it is recorded as a negative polarity event $p_{i}=-1$. The polarity of an event can be defined by the following mathematical expression:

(1) $$p=\left\{\begin{array}{l} +1,\log I(x,y,t+\Delta t)-\log I(x,y,t)>\Delta I\\ -1,\log I(x,y,t+\Delta t)-\log I(x,y,t)<\Delta I \end{array}\right.$$

where logI(x,y,t) represents the logarithmic light intensity of the pixel with coordinate (x,y) at moment t, and Δ*I* represents the light intensity change threshold.

### 3.2. Event Stream Denoising Based on Spatio-Temporal Density and Temporal Analysis

**Figure 3:**
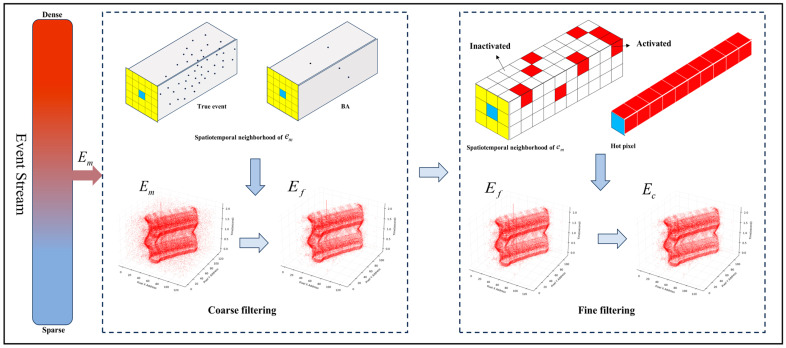
Proposed event stream denoising framework based on spatio-temporal density and time sequence analysis, where *E_m_* represents the unprocessed event stream, *E_f_* represents the event stream that enters fine filtering after coarse filtering, and *E_c_* represents the processed event stream. Blue for newly arrived events, yellow for their spatial neighborhoods, and red for pixels activated.

Valid events are usually triggered by the motion of an object or by a change in lighting. Most of these activated pixels are adjacent to each other. Due to the handshake circuit design of the DVS, events in the same row of pixels have the same timestamp, while the difference in event times between pixels in neighboring rows is very small, usually measured in nanoseconds. The number of events generated by background activity (BA) in a given spatio-temporal region is usually much lower than the number of events generated by actual object motion. Since hot pixels are continuously activated and output events due to their inability to be properly reset, the location of occurrence is random, and thus the number of events occurring on hot pixels is usually much higher than that on neighboring pixels within a given spatio-temporal region. Based on the analysis of BA noise and hot pixel noise generation principle and spatio-temporal distribution characteristics, we are more concerned about how to target the removal of different noises. Figure 3 shows the framework of the denoising algorithm.

#### 3.2.1. Spatio-Temporal Density-Based Denoising of Event Stream

**Figure 4:**
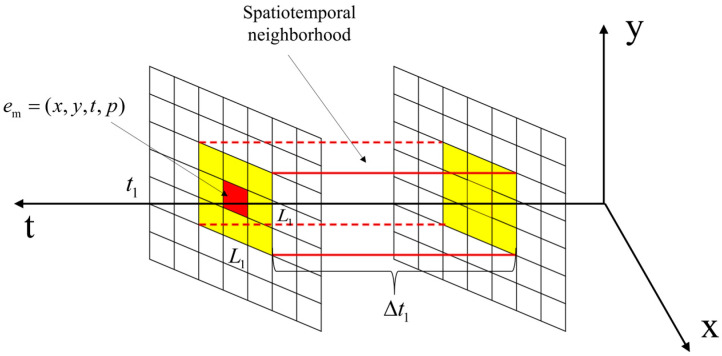
Spatio-temporal neighborhood of *e_m_*.

To improve the performance and robustness of the denoising algorithm, the information of temporal and spatial neighborhoods is comprehensively utilized in the coarse filtering stage to maximize the extraction of useful information and the suppression of noise, to make it more suitable for applications in various complex scenarios. The temporal and spatial neighborhoods are set for each event. The red region in Figure 4 is the newly arrived event $e_{m}$, which is located in the center of the spatial neighborhood of size $L_{1}\times L_{1}$ ($L_{1}$ is an odd number), the temporal neighborhood is $\left(t_{1}-\Delta t_{1},t_{1}\right)$, and $N_{local}$ denotes the number of events in the spatial and temporal neighborhoods $\Omega_{\Delta t_{1}}^{L_{1}}$. The yellow region is the spatial neighborhood of $e_{m}$.

All events in the spatio-temporal neighborhood are counted, and the density of the spatio-temporal neighborhood is calculated. If the event spatio-temporal density is greater than a threshold, it is considered a valid event; if it is less than the threshold, it will be considered noise. The spatio-temporal density is defined as follows:

(2) $$D(e_{m})=\frac{N_{local}(e_{m})}{{L_{1}}^{2}\cdot \Delta t_{1}}$$

where $N_{local}(e_{m})$ is the total number of events in the spatio-temporal neighborhood of $e_{m}$.

The noise identification formula of $e_{m}$ is as follows:

(3) $$e_{m}=\left\{\begin{array}{l} Noise,\;if\;D(e_{m})<DT\\ Vaild,\;if\;D(e_{m})\geq DT \end{array}\right.$$

where DT is a preset threshold for distinguishing BA noise from valid events.

DT can be dynamically adjusted to adapt to different noise environments according to the characteristics of the actual scene. The regularization of the number of events is achieved by dividing the number of events in the neighborhood by the volume of the neighborhood. This means that the density value reflects the level of event activity per unit volume regardless of the size of the neighborhood. Evaluating event densities from a relative perspective prevents the results from being unfairly affected by changes in neighborhood size. Comparing the spatio-temporal density of each event with the density threshold effectively filters out the BA noise and retains the valid events in the coarse filtering results for fine filtering.

#### 3.2.2. Event Stream Denoising Based on Timing Analysis

Event cameras upload a fixed format event when the brightness value of a pixel at a location changes, so pixels can be categorized into active and inactive states during operation. A hot pixel is continuously activated at a fixed location and there may be real events or BA noise in the neighboring pixels. The spatio-temporal density approach does not provide effective removal of hot noise. Therefore, it is necessary to fine filter the event stream after coarse filtering. The hot noise filtering method used by Feng assumes that in an ideal environment, there is no BA noise in the hot noise spatio-temporal neighborhood, or only a very small amount of noise exists. In the experimental process of Section 4, it is found that all BA noise often cannot be effectively filtered out in a very dense noise environment, and at this time the accuracy of the denoising algorithm for hot noise will show a significant decrease. Therefore, we use the denoising algorithm based on time sequence analysis to remove hot noise.

For the coarsely filtered events, a new spatio-temporal neighborhood $\Omega_{\Delta t_{2}}^{L_{2}}$ is constructed. The working states of the event pixels as well as the neighboring pixels are sampled within $\Delta t_{2}$, with 1 representing a pixel activated and 0 representing a pixel inactive, and a Boolean time series is obtained at the end of the sampling. The working state S∈{1,0} of the pixel is recorded at the sampling time point $t_{k}$, then the event pixel is obtained as a time sequence $\left\{S_{i}\left(t_{0}\right),S_{i}\left(t_{1}\right),S_{i}\left(t_{2}\right),\cdot \cdot \cdot \cdot ,S_{i}\left(t_{f\cdot \Delta t_{2}-1}\right)\right\}$. For each spatial neighborhood pixel of pixel i, the corresponding time series can be obtained $\left\{S_{j}\left(t_{0}\right),S_{j}\left(t_{1}\right),S_{j}\left(t_{2}\right),\cdot \cdot \cdot \cdot ,S_{j}\left(t_{f\cdot \Delta t_{2}-1}\right)\right\}$, $j\in N\left(i\right)$, f is the sampling frequency.

Hot pixels exhibit persistent activation states, and neighboring pixels usually have a much lower proportion of activated states than hot pixels. The DC component of a time series after discrete Fourier transform indicates the average level of the time series, and for Boolean time series, this value reflects the proportion of 1 states in the time series. Hot noise is removed by comparing the DC component of an event stream pixel $DC_{i}$ with the average DC component of its neighboring pixels $\bar{DC_{j}}$. Although a certain amount of hot noise can be filtered out by directly comparing the values of $DC_{i}$ and of $\bar{DC_{j}}$, in order to enable the noise reduction algorithm to be more flexible, a threshold value is introduced θ. The noise identification formula of $e_{m}$ is as follows.

(4) $$e_{m}=\left\{\begin{array}{l} Noise,\;if\;DC_{i}\geq \bar{DC_{j}}+\theta \\ Vaild,\;if\;DC_{i}<\bar{DC_{j}}+\theta \end{array}\right.$$

In the fine filtering algorithm, the choice of threshold θ is very important and determines the sensitivity and specificity of the determination. A threshold that is too low may result in too many valid events being misclassified as noise, while a threshold that is too high may result in failure to detect true hot noise. Therefore, it may be necessary to experiment and analyze specific data to adjust the threshold value to achieve the best denoising performance.

### 3.3. An Adaptive Overlap-Based Event Stream Visualization Method

Motion state changes and scene variations during event camera operation can lead to an uneven amount of event information per unit of time. Compared with visualization methods that simply fix the number of events or a fixed time window, the overlapping cumulative event stream visualization method optimizes the visual presentation of a dynamic scene by sharing events between consecutive frames and achieves an increase in the information richness of each frame while maintaining a high frame rate. This, to some extent, enables the event frames to ensure the continuity of event information despite the uneven amount of event information. However, the fixed overlap parameter is difficult to apply to various complex scenes.

In this paper, an adaptive overlap time strategy is proposed to dynamically adjust the overlap time according to the change in event density. The adaptive strategy can make it possible to increase the overlap to capture more details when the scene changes faster and decrease the overlap to reduce redundancy when the scene changes slower. A baseline event density threshold is defined, the number of events in the *i*-th time slice is counted, and the time at which the *i*+1-th time slice overlaps the previous frame is determined by Equation (5). Since the dynamic scene changes in different degrees, it is difficult to use a fixed step size to adjust the overlap time, so the step size value is based on the event density difference. Define the time slice length and overlap time *O*. The expression for adaptive overlap of events is as follows.

(5) $$O_{i+1}=O+sign(d-D_{i})\cdot (k\cdot \left|d-D_{i}\right|)$$

where $D_{i}$ is the number of events in the i-th time-slice length, k is the step adjustment factor, and $O_{i+1}$ is the time at which the i+1-th time-slice overlaps the previous frame.

**Figure 5:**
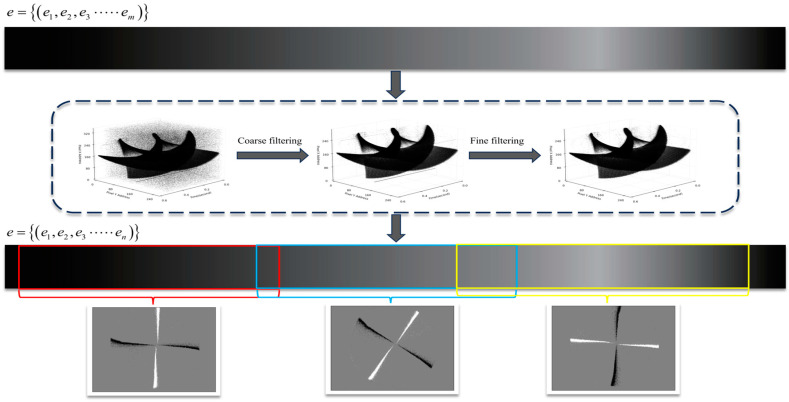
Denoising and visualization process, The event stream passes through the cascaded filter, filtering out n-m events. Three color boxes represent three event frames. The overlapping areas between the boxes represent the events that overlap with the previous frame.

The noise processing and visualization process is shown in Figure 5. A set of event streams with uneven density distribution are filtered by coarse fine filtering, and then clear and coherent event frames are obtained based on the adaptive overlapping method. The event stream’s color represents the events’ density (black is the densest, and white is the sparsest). Different colors represent the events mapped to each event frame. The adaptive overlap method ensures flexible event quantity mapping and event overlap for each event frame.

## 4. Experiment and Evaluation

The experiments in this chapter are divided into three sections. Section 4.1 captures a real scene using the Davis346 event camera and denoises the dataset using different denoising algorithms. Section 4.2 adds random noise to multiple public AER datasets and compares the denoising results of different denoising algorithms. Section 4.3 evaluates the visualization algorithms. Section 4.1 and Section 4.2 use the filters of NN2, Khodamoradi, and Feng for comparison with our algorithm. The computer processor is an Intel(R) Core(TM) i7-9700 CPU at 3.3 GHz, 8 GB of RAM, and the operating system is Windows 10 64-bit.

### 4.1. Event Stream Denoising Experiments in Real Scenes

In this experimental phase, we use a Davis346 with a spatial resolution of 346 × 260. Because event cameras are very good at capturing fast-moving objects, in Scene I we use it to capture a fast-moving soccer ball, which goes from slow to fast until it is completely thrown. In Scene II, we use Davis346 to record a black and white disk rotating at high speed, and the rotation speed of the disk changes from fast to slow with time. Due to the experimental scene with more factors interfering, the event stream output by Davis346 contains a lot of noise.

Since there is no base truth value of comparable events from real-world data, NIR (Noise in Real) and RIN (Real in Noise) are used to evaluate the denoising performance of different algorithms in real-world scenarios [25]. NIR measures the number of noisy events that are erroneously retained after denoising, and smaller NIR values indicate that the denoising algorithm is more effective in removing noise.

(6) $$NIR=\sum \delta (x-x_{i},y-y_{i},t-t_{i})\;e_{i}(x_{i},y_{i},t_{i})\in \Lambda_{PE}{\;\mathrm{and}\;P}_{ei}\leq P_{ARE}$$

where $\Lambda_{PE}$ is the set of events delivered during the cycle, and $P_{ARE}$ is the average true event probability of the BA computed over events with no target motion region.

RIN measures the number of real events that were mistakenly deleted; smaller RIN values mean that more real and valid events were retained while removing noise.

(7) $$RIN=\sum \delta (x-x_{i},y-y_{i},t-t_{i})\;e_{i}(x_{i},y_{i},t_{i})\in \Lambda_{PE}{\;\mathrm{and}\;P}_{ei}>P_{ARE}$$

where $\Lambda_{FE}$ is the set of filtered events in the cycle.

**Figure 6:**
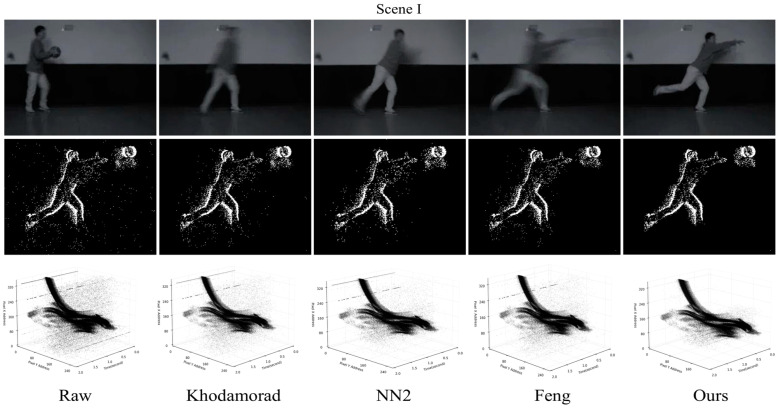

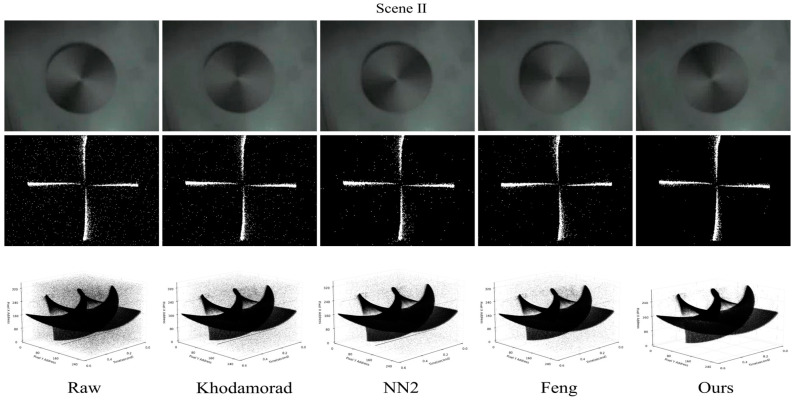
Event denoising results. The first row is the output of the frame-based camera, and the second and third rows are the 2D maps and spatio-temporal images of the original event stream and the event stream after denoising by different methods.

In the proposed algorithm, we have multiple parameters and calibrate some of them based on the data used. The values of the parameters should vary depending on the application field and be minimized to achieve more accurate denoising. The method used in this paper uses the parameters $L_{1}/\Delta t_{1}=7/3$ ms, $L_{2}/\Delta t_{2}=5/6$ ms, DT = 10, and θ=15. The parameter in the Feng filter is set to L/Δt=5/5 ms, and the parameter in the Khodamoradi filter is set to 2 ms. It should be noted that in the algorithm proposed in this paper, there is a minimum spatio-temporal neighborhood setting L/Δt=3/1 ms. This parameter implies the strictest noise discrimination, although this does not apply to the vast majority of scenarios. We adjusted the parameters with the minimum spatio-temporal neighborhood as a benchmark, so the choice of algorithmic parameters for each scenario is based on the results of multiple experiments. Moreover, in the choice of parameters for the three comparative algorithms, we used the default parameters provided in the paper. Figure 6 shows the denoising results of the different algorithms for two scenarios, Table 1 shows the change in the number of events before and after denoising for both scenarios, as well as the noise evaluation based on *RIN* and *NIR*.

In the denoising results shown in Figure 6 and Table 1, our algorithm filters out 19.09% of the events in Scene I, which is higher than 17.34% for NN2, 14.87% for the Feng filter, and 8.66% for the Khodamoradi filter. In scene II, our algorithm filters out 19.04% of the events, higher than 16.09% for NN2, 13.9% for the Feng filter, and 9.57% for the Khodamoradi filter. The RIN and NIR values of this paper’s algorithm in Scene I do not show a significant advantage due to the discontinuous and irregular distribution of the background noise region, but the 2D plots and spatio-temporal images show that our algorithm filters out more noise compared to other algorithms. This suggests that the reliability of RIN and NIR denoising assessment based on manual selection of background noise regions still needs to be enhanced.

### 4.2. Denoising the AER Datasets

Denoising the event stream output by the event camera as a preprocessing method can facilitate the subsequent obtaining of clearer event frames. To verify the denoising performance of our proposed algorithm in different noise environments, we designed the following experiments. Events in the original AER dataset are treated as valid events, and a certain percentage (10%, 20%, 50%, 100%, 150%, and 200%) of noise (BA noise and hot noise) is added. In the denoising experiments of the AER dataset, we use the event denoising precision (EDP) and the event Signal to Noise Ratio (ESNR) to measure the accuracy of the denoising algorithm in removing noise [26]. EDP is a measure of whether the denoising algorithm effectively filters out the noise in the event stream, and the larger the value, the better the denoising effect. ESNR is a measure of the quality of the event stream, and a high ESNR value denotes a lower noise level and a higher quality of the event stream.

(8) $$EDP=\frac{N_{effective}}{N_{total}}$$

where $N_{\mathrm{effective}}$ is the total number of valid events in the event stream and $N_{\mathrm{total}}$ is the total number of events in the event stream.

(9) $$ESNR=10\mathrm{lg}\left(\frac{N_{noise}}{N_{\mathrm{effective}}}\right)$$

where $N_{noise}$ is the total number of noise events in the event stream.

Experiments were performed on the public AER datasets N−MNIST [35], CIFAR10−DVS [36], and MNIST−DVS [37]. The MNIST−DVS dataset was zoomed from the original MNIST dataset image to three scales (Scale 4, Scale 8, and Scale 16) and displayed in slow motion on an LCD monitor. Event-based sensors were then used to record the number of movements. The MNIST−DVS dataset with a scale of 4 was used in the experiments. CIFAR10−DVS is an event dataset for object classification. The 10,000 frame-based images from CIFAR-10 are converted into 10,000 event streams by DVS with a resolution of 128 × 128 pixels, and the conversion is achieved by using Repetitive Closed Loop Smoothing (RCLS) motion on the frame-based images. The N−MNIST dataset is a spiked version of the MNIST dataset, containing the same samples as the original MNIST dataset and captured at the same visual scale (28 × 28 pixels) as the original MNIST dataset. The N-MNIST dataset is captured by mounting the ATIS sensor on a motorized gimbal and causing the sensor to move while viewing the MNIST example on the LCD monitor.

**Figure 7:**
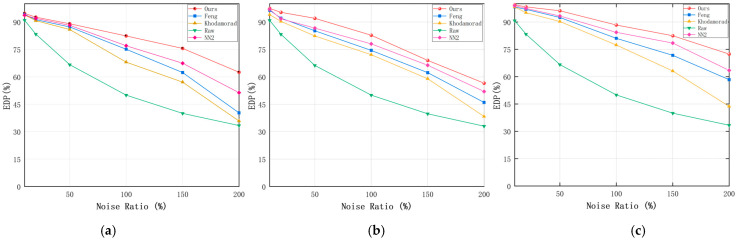
EDP comparison of filter algorithm and our algorithm on different datasets. (**a**) CIFAR10-DVS; (**b**) MNIST-DVS; (**c**) N-MNIST.

**Figure 8:**
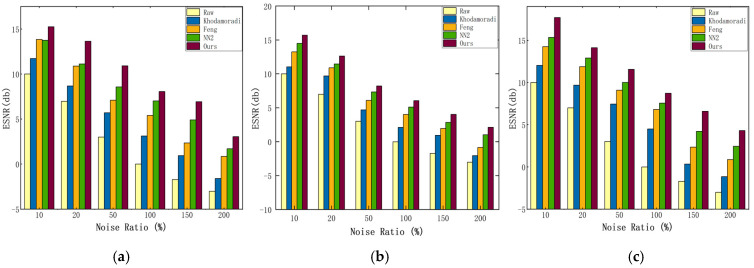
ESNR comparison of filter algorithm and our algorithm on different datasets. (**a**) CIFAR10−DVS; (**b**) MNIST−DVS; (**c**) N−MNIST.

In this section of experiments, the method of this paper uses the parameters $L_{1}/\Delta t_{1}=7/3$ ms, $L_{2}/\Delta t_{2}=5/6$ ms, DT = 10, and θ=15. The parameter in the Feng filter is set to L/Δt=7/5 ms and the Khodamoradi filter parameter is set to 2 ms. Figure 7 and Figure 8 show the ESNR vs. EDP for the three datasets N−MNIST, CIFAR10−DVS, and MNIST−DVS.

As shown in Figure 7 and Figure 8, when the noise is sparser, the denoising methods may be more likely to accurately restore the true event distribution, when the difference in the denoising effect of different algorithms is small. However, when the noise is denser, the denoising methods may be more challenged, and the EDP and ESNR values show a larger magnitude decrease.

As can be seen from Table 2 and Table 3, there is a positive correlation between EDP and ESNR, indicating that ESNR and EDP can reflect the quality of the event stream well. The performance of Feng’s filter shows a significant degradation after the addition of dense noise, which is because, in a dense noise environment, BA noise may show some spatial-temporal correlation between them, and BA noise may also show some spatial-temporal correlation between them and the real events, and at this time, it is difficult to discriminate the noise, a lot of BA noise is retained after coarse filtering, which affects the denoising accuracy of the fine filtering. The Khodamorad filter can efficiently filter out most of the noise according to the spatio-temporal correlation in a sparse noise environment, and when the noise is denser, the denoising accuracy decreases seriously and the hot noise is not effectively removed. The denoising effect of NN2 shows better robustness in a more dense noise environment. However, NN2 cannot effectively remove hot noise, because hot noise has a certain spatial-temporal correlation and the db value is higher than BA noise, so it is difficult to distinguish it from real events during testing.

Our algorithm shows advantages in EDP and ESNR metrics, and it is noteworthy that the reduction in EDP and ESLR is the same in different proportions of noise environments, and there are no problems with significant reduction due to dense noise environments. Due to the regularization of the spatio-temporal neighborhood in the coarse filtering, the spatio-temporal density is not unfairly affected by the variation of the neighborhood size, and the vast majority of BA noise is filtered out in the coarse filtering stage. This avoids the drawback of counting the number of events in the dense noise environments based on the spatio-temporal correlation theory, and the method based on the temporal analysis in the fine filtering ensures that the thermal noise can be accurately filtered out even in the presence of the BA noise. However, more complex algorithms imply that they will occupy more memory and require more computation. In our experiments, we found that our proposed algorithm does not show an advantage in time, which is due to the fact that in fine filtering, the computation is increased due to the temporal analysis of the pixels where each event is located.

### 4.3. Event Stream Visualization

**Figure 9:**
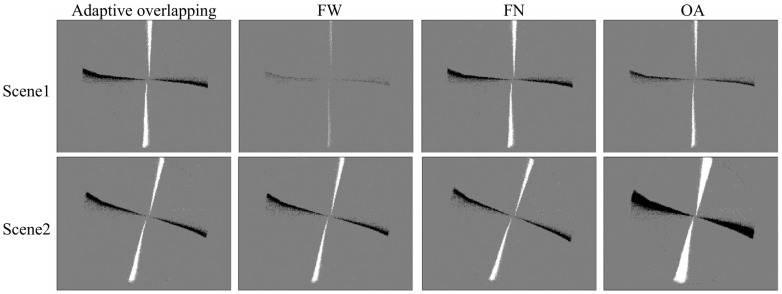
Visualization results for black and white turntable. The time window is 10 ms, and the number of events are 8000, O=5 ms, and *k* = 0.001.

**Figure 10:**
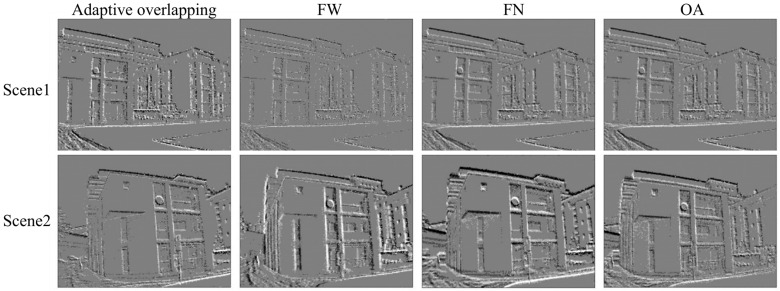
Visualization results for urban dataset. The time window is 10 ms, and the number of events are 12,000, O=5 ms, and *k* = 0.001.

To verify and analyze the effect of event stream visualization, this section experiments on a real dataset as well as a public dataset. The real dataset uses the fast-rotating black-and-white turntable recorded in Section 4.1. As shown in Figure 9, the turntable rotates at a slower speed in Scene 1, and the event stream is sparse. The turntable rotates faster in Scene 2. The time stream is denser. The public event dataset was acquired by the Sensor Laboratory at ETH Zurich using a DAVIS 240C model event camera [38], with the urban part as the scene for the experiment as shown in Figure 10. In Scene 1, the motion state of the camera is smoother and the event stream is sparse. In Scene 2, the camera motion state is more violent and the event stream is denser. Table 4 shows the number of events in different scenes as well as time. The experiments compare four event frame formation methods: adaptive overlapping, fixed time window (FW), fixed number of events (FN), and overlapping accumulation (OA). The frame rate, the average number of events, and the variance of the number of events obtained by different visualization methods can reflect to a certain extent the uniformity of the information contained in the event frames, regardless of the intensity or slowness of the operation. Therefore, we use the above three indicators to evaluate the event frames obtained by different methods, as shown in Table 5 and Table 6.

In this section of experiments, the method of this paper uses the parameters O=5 ms, k=0.001. The results of the event flow visualization are shown in Figure 9 and Figure 10, where black represents negative events and white represents positive events.

Since scene changes as well as motion state changes lead to uneven distribution of events in time, accumulating events according to a fixed time window results in event frames containing different amounts of information, blurring of event edges when motion is smooth, and severe shadowing of object edges due to event accumulation when motion is violent. The overlapping accumulation method can both increase the frame frequency and ensure the same amount of event information as that of a fixed time window. However, when the density of the event stream changes, a single overlapping strategy can lead to redundant or incoherent information. Fixing the number of events ensures a consistent amount of information, but when the motion is very smooth, it leads to long output frames containing a large number of outdated events, thus losing the low-latency advantage of event cameras. As shown in Table 5 and Table 6, the fixed time window method has a fixed output frame rate and the largest event variance. A fixed output frame rate implies a stable number of output frames, but a large event variance indicates a large difference in the amount of information between frames. The overlapping accumulation method has the largest output frame rate and the second-largest event variance. The maximum output frame rate means that more motion details can be captured, but it also contains a large amount of duplicate content. On the other hand, the fixed number of events method has the smallest event variance, but also the lowest frame rate. The adaptive overlapping approach did not show a clear advantage in one metric or the other but ensured a higher frame rate output while avoiding too much information variance between frames.

## 5. Conclusions

In this paper, an event stream denoising method based on spatio-temporal density and temporal sequence analysis is proposed. Based on the working principle of the event camera and the noise distribution characteristics, the noise is categorized into BA noise and hot noise, and the event stream is sequentially coarse filtered and fine filtered. We pay more attention to the optimization and improvement of the robustness of the denoising algorithm, so the parameter selection and the influence of the residual BA noise are taken into account in different filtering stages. The spatio-temporal neighborhood regularization and the free choice of threshold make our algorithm applicable to various noise environments. We use EDP and ESNR to evaluate the denoising effect of the AER dataset, and NIR and RIN to evaluate the denoising effect of the real scene. In addition, an event stream visualization method based on adaptive overlapping is proposed to validate the visualization algorithm by selecting some scenes in two datasets based on adaptive overlapping events of camera motion state changes or scene changes.

In future research and development work, we will continue to optimize our algorithm, focusing on further reducing the complexity of our algorithm so that it can be used in tight resource environments, and we will explore ways to integrate denoising techniques into real-time systems.

## Figures and Tables

**Table 1:** Noise evaluation of Scene I and Scene II.

Scene	Index	Raw Data	Khodmradi	NN2	Feng	Ours
I	Number of events	196,232	179,243	162,203	167,043	158,882
	RIN	/	7029	5330	5208	5294
	NIR	/	29,445	8401	8790	8328
II	Number of events	731,014	661,027	609,112	629,428	591,840
	RIN	/	12,936	6230	8979	5022
	NIR	/	134,502	49,389	72,103	32,357

**Table 2:** Comparison of the performance of different denoising algorithms on EDP.

Dataset	Algorithm	Noise Ratios					
		10%	20%	50%	100%	150%	200%
CIFAR10−DVS	Raw	90.95	83.33	66.67	50	39.99	33.33
	Khodamorad	93.92	90.76	85.88	68.05	57.13	35.82
	NN2	93.76	91.99	88.21	77.02	67.45	51.3
	Feng	94.21	91.29	87.33	75.09	62.35	40.32
	Ours	94.73	92.69	89.03	82.41	75.57	62.51
MNIST−DVS	Raw	90.92	83.27	66.27	50	39.76	33.03
	Khodamorad	94.02	90.35	82.37	72.12	58.96	38.32
	NN2	96.96	91.75	86.63	78.02	66.34	51.94
	Feng	96.43	92.17	85.2	74.48	62.33	45.99
	Ours	97.47	95.34	92.04	82.79	69.01	56.53
N−MNIST	Raw	90.74	83.31	66.61	50	39.96	33.33
	Khodamorad	98.22	95.16	90.26	77.34	63.11	43.69
	NN2	98.95	97.46	93.26	84.32	78.45	63.44
	Feng	98.23	96.93	92.46	81.03	71.69	58.37
	Ours	99.44	98.31	96.15	88.36	82.45	72.39

**Table 3:** Comparison of the performance of different denoising algorithms on ESNR.

Dataset	Algorithm	Noise Ratios					
		10%	20%	50%	100%	150%	200%
CIFAR10−DVS	Raw	10	6.98	3.01	0	−1.72	−3.02
	Khodamorad	11.72	8.68	5.7	3.12	0.94	−1.6
	NN2	13.74	11.12	10.92	8.06	6.93	3.04
	Feng	13.85	10.88	7.09	5.41	2.34	0.86
	Ours	15.26	13.64	10.92	8.06	6.93	3.04
MNIST−DVS	Raw	10	6.98	3.01	0	−1.72	−3.02
	Khodamorad	11.02	9.68	4.7	2.12	0.94	−2.06
	NN2	14.49	11.46	7.34	5.11	2.84	1.02
	Feng	13.25	10.88	6.09	4.01	1.94	−0.86
	Ours	15.7	12.64	8.2	6.06	4.03	2.14
N−MNIST	Raw	10	6.98	3.01	0	−1.72	−3.02
	Khodamorad	12.02	9.68	7.45	4.49	0.34	−1.16
	NN2	15.33	12.91	10.01	7.55	4.21	2.44
	Feng	14.25	11.88	9.09	6.81	2.34	0.86
	Ours	17.7	14.12	11.56	8.72	6.59	4.3

**Table 4:** Parameters for different scenes.

Dataset	Scene	Event Number	Time (s)
Turntable	1	184,402	1.2
	2	284,125	1.15
Urban	1	384,246	0.9
	2	620,143	1.1

**Table 5:** Performance comparison of four visualization methods for black and white turntable dataset.

	Algorithm	Evaluation Metrics		
		Frame Rate (fps)	Average Number of Events	Variance
Scene 1	FW	199.97	2234.2	1,523,975.02
	FN	44.61	7993.11	1973.29
	OA	322.94	3749.12	1,493,276.06
	Ours	157.49	7319.86	1,052,987.34
Scene 2	FW	196.32	7294.32	1,347,349.45
	FN	54.13	7998.21	447.96
	OA	380.17	1492.21	1,283,601.73
	Ours	165.49	6971.13	923,839.03

**Table 6:** Performance comparison of four visualization methods for urban dataset.

	Algorithm	Evaluation Metrics		
		Frame Rate (fps)	Average Number of Events	Variance
Scene 1	FW	195.38	5825.63	3,413,492.31
	FN	40.22	11,982.33	4216.03
	OA	375.94	11,049.12	3,013,484.39
	Ours	169.49	9932.36	2,269,272.26
Scene 2	FW	192.91	13,438.93	5,312,380.2
	FN	49.43	11,970.21	10,235.27
	OA	417.68	10,492.75	4,526,249.37
	Ours	201.33	9523.19	3,028,346.12

## Data Availability

The data used to support the research results of this paper can be obtained from the corresponding author upon request.
